# Stochastic sensing of Angiotensin II with lysenin channels

**DOI:** 10.1038/s41598-017-02438-0

**Published:** 2017-05-26

**Authors:** Nisha Shrestha, Sheenah L. Bryant, Christopher Thomas, Devon Richtsmeier, Xinzhu Pu, Juliette Tinker, Daniel Fologea

**Affiliations:** 1 Department of Physics, Boise State University, Boise, ID 83725 USA; 2 Biomolecular Sciences Graduate Program, Boise State University, Boise, ID 83725 USA; 3 Biomolecular Research Center, Boise State University, Boise, ID 83725 USA; 4 Department of Biology, Boise State University, Boise, ID 83725 USA

## Abstract

The ability of pore-forming proteins to interact with various analytes has found vast applicability in single molecule sensing and characterization. In spite of their abundance in organisms from all kingdoms of life, only a few pore-forming proteins have been successfully reconstituted in artificial membrane systems for sensing purposes. Lysenin, a pore-forming toxin extracted from the earthworm *E. fetida*, inserts large conductance nanopores in lipid membranes containing sphingomyelin. Here we show that single lysenin channels may function as stochastic nanosensors by allowing the short cationic peptide angiotensin II to be electrophoretically driven through the conducting pathway. Long-term translocation experiments performed using large populations of lysenin channels allowed unequivocal identification of the unmodified analyte by Liquid Chromatography-Mass Spectrometry. However, application of reverse voltages or irreversible blockage of the macroscopic conductance of lysenin channels by chitosan addition prevented analyte translocation. This investigation demonstrates that lysenin channels have the potential to function as nano-sensing devices capable of single peptide molecule identification and characterization, which may be further extended to other macromolecular analytes.

## Introduction

More than two decades of studies focused on investigating the minute changes in ionic currents through single synthetic and natural nanopores upon their interaction with analyte molecules have paved the way for applications such as single molecule identification and characterization, biosensing, diagnosis, and drug discovery^1–6^. The ultimate goal of DNA sequencing prompted scientists to focus on using nanopores for nucleic acids studies^1, 7–10^. Nonetheless, there is an increasing interest in using nanopore-based technologies for peptide sensing and characterization^11–16^ which is fueled by the prospect of rapid, reliable, and cheap peptide identification, quantification, and even sequencing. This particular interest in peptide detection is amplified by their potential involvement in the onset of cancer, neurodegenerative disorders, and infections^17–19^, as well as established correlations between their expression levels and diseases^20–22^. While synthetic nanopores are considered scalable and more robust both chemically and mechanically, their production with desired dimensions and sub-nanometer precision is still a challenging task^23–25^. In contrast, nanopores of biological origin present outstanding structural repeatability at the atomic level and are more amenable to chemical modification and sophisticated bio-engineering procedures intended to significantly extend their sensing capabilities^6, 26–28^. Since the first use of the α-hemolysin (α-HL) nanopore for successful translocation of DNA molecules^29^, only a few other biological channels have been proposed and used as stochastic sensors for nucleic acids and/or peptides. The most used among these are aerolysin^8, 14^, the phi29 viral motor^6, 11, 30, 31^, and the MspA channel^7, 32–34^. These reports identified several limitations of the use of biological nanopores for peptide translocation studies with regards to the limited size of the analyte, undesired interactions between the translocated molecules and the channels, or the requirement of labor-intensive steps for nanopore preparation and reconstitution into artificial membrane systems. In our endeavor to minimize these limitations, we investigated the use of lysenin as an alternative nanopore for peptide translocation studies. Lysenin is a pore-forming toxin extracted from the coelomic fluid of the earthworm *E. fetida*, which self-assembles into large nonameric channels in artificial and natural lipid membranes containing sphingomyelin^35–39^. Lysenin channels are in the open state at negative voltages^40^ and are stable for a large range of electrolyte concentrations and pH^41^. The recently deciphered structure of the assembled lysenin channel reveals a long β-barrel (~9–11 nm) with a pore ~2.5 nm in diameter and no apparent vestibular structures^35, 36^. The wide pore opening may facilitate translocation of large molecules, thus presenting potential sensing capabilities. Recent attempts to translocate ssDNA molecules through the wild type channel failed^36^, presumably owing to strong electrostatic repulsion between the channel and negatively charged polymers. However, the same study reports that a mutant constructed by replacing five negatively charged amino acids with neutral and positively charged ones apparently allows for the capture and translocation of ssDNA molecules^36^. To test the hypothesis that wild type lysenin channels may accommodate the passage of large molecules, we focused on investigating translocation of cationic peptides. In this respect, we chose human angiotensin II (Ang II) as a model analyte which is a short octameric peptide hormone bearing a fractional positive charge at neutral pH. Experiments that employed single lysenin channels inserted into artificial lipid membranes revealed distinct electronic signatures of analyte-nanopore interactions, which we classified as putative translocations or collisions based on previous interpretations of similar experimental data. Liquid Chromatography-Mass Spectrometry (LC-MS) analysis identified Ang II in samples collected after employing large populations of lysenin channels and extended time scales, therefore providing proof of translocation. In addition, application of reverse transmembrane electric fields or irreversible blockage of the lysenin’s conducting pathway by chitosan addition prevented the LC-MS detection of the translocated analyte.

## Results and Discussions

### Ang II interaction with lysenin channels ellicits transient changes in the ionic current

**Figure 1:**
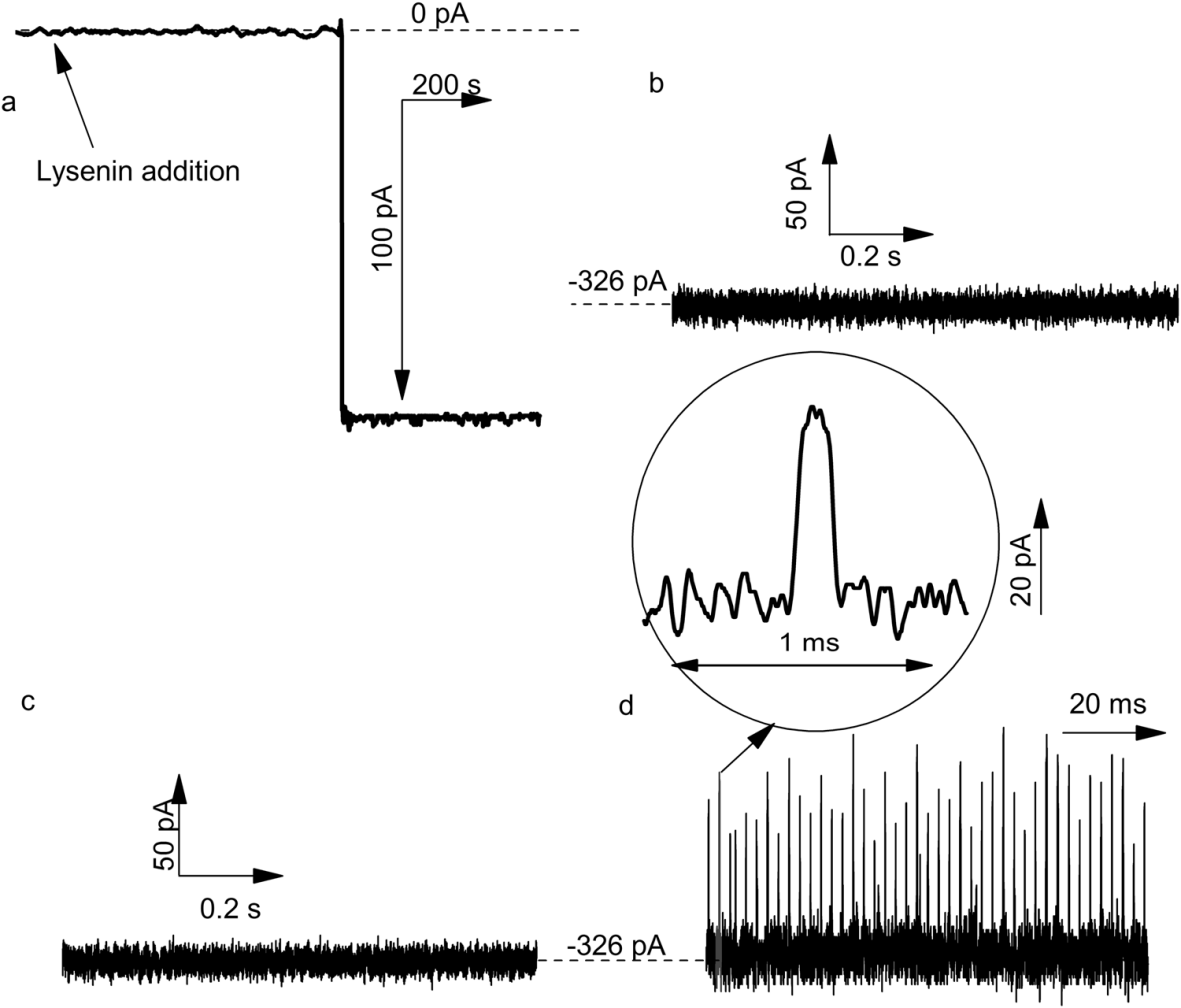
Interaction of Ang II with single lysenin channels inserted into lipid membranes bathed by 1 M KCl solutions buffered with 10 mM Tris and 1 mM EDTA at pH 6.9. (**a**) Insertion of a single channel in the bilayer membrane was observed as a step change in the ionic current at −60 mV transmembrane potential (sampling time 1 s, 1 kHz low pass hardware filter, and 10 Hz low pass software filter). No transient changes in the ionic current established through two open lysenin channels were observed at −80 mV when: (**b**) no Ang II was added to the solutions, and (**c**) Ang II was added to the *trans* reservoir. (**d**) Addition of Ang II to the *cis* reservoir yielded multiple transient changes in the ionic current, indicative of interactions between the channel and peptides. The traces shown in panels (**b**–**d**) have been recorded with a sampling time of 4 μs and a 10 kHz low pass hardware filter.

The core experimental setup for the analysis of interactions between Ang II and single lysenin channels, common for macromolecule translocation studies, is detailed in the methods section. The insertion of a single lysenin channel into the membrane was indicated by a steady open current of ~−122 pA at −60 mV bias potential (Fig. 1a), after which the solution in the *cis* reservoir was exchanged with lysenin-free electrolyte to prevent further insertions. However, more channels may insert after buffer exchange since the formation of a pre-pore attached to the membrane is a condition for channel oligomerization^36^. Although we determined no changes in the characteristic electronic signatures derived from single channel measurements for up to six inserted nanopores (after which the electric noise may become significant and prevent accurate analysis), all translocation experiments on single channels consistently comprised two lysenin nanopores assembled into the lipid membrane.

In the absence of Ang II peptide analyte added to the external solutions, the ionic current trace recorded through two channels at −80 mV and high temporal resolution (4 µs sampling time) indicated the absence of any transient change in the open current trace (Fig. 1b) and low noise (<2.6 pA RMS at 10 kHz bandwidth). Similarly, no transient changes in the open current were observed after the addition of 1 μg mL^−1^ Ang II into the *trans* (headstage) reservoir for otherwise identical experimental conditions and time scale (Fig. 1c), suggesting that the particular direction of the electric field prevented any nanopore-analyte interactions. In contrast, peptide addition into the *cis* reservoir at −80 mV transmembrane potential yielded frequent and short transient changes in the ionic current (Fig. 1d), indicative of peptide interactions with the open channel^13–16, 42–46^.

### The two major types of recorded events may be classified as translocations or collisions

**Figure 2:**
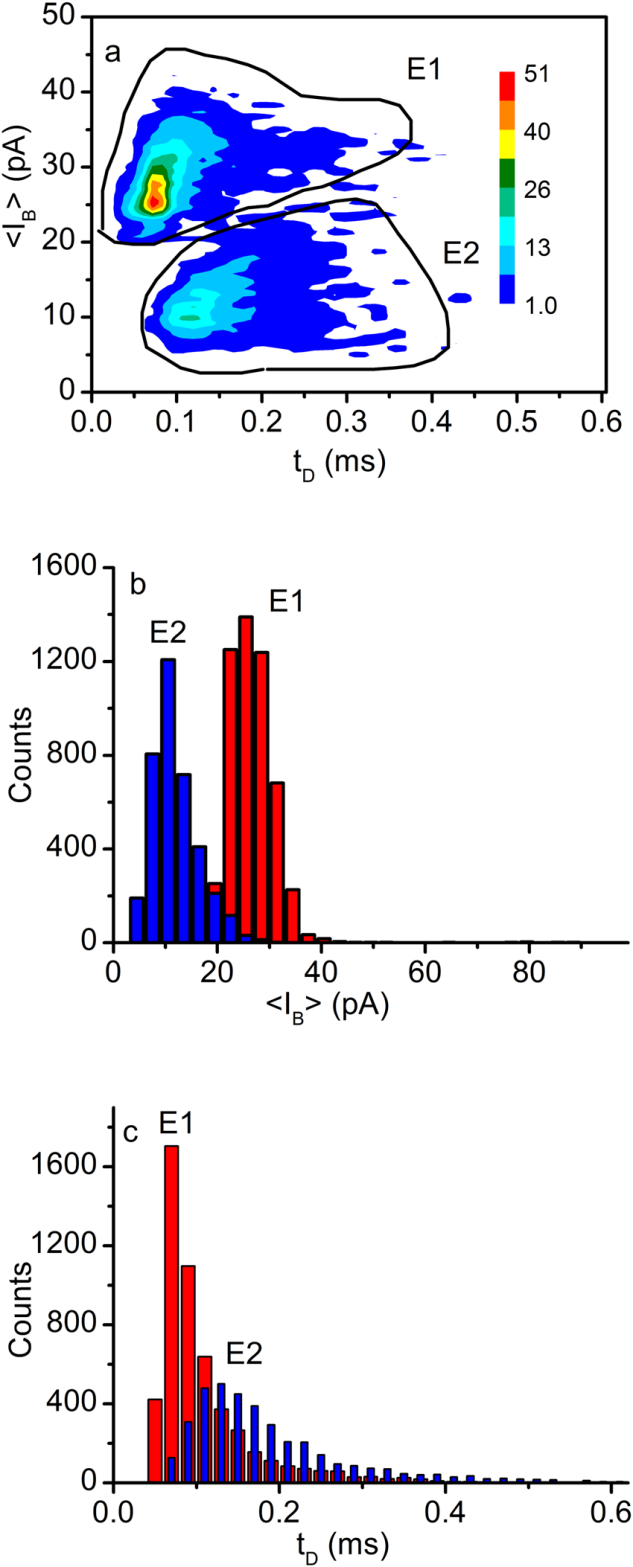
Analysis of the blockage events recorded after Ang II addition to the *cis* reservoir at −80 mV transmembrane potential. (**a**) The density plot shows two well-defined clusters (named E1 and E2), which allowed separate analysis of each individual cluster. The color indicates the density in accordance to the inserted scale. (**b**) The <I_B_> histograms (bin width 3 pA) present peaks at ~26 pA (E1 events) and ~10 pA (E2 events). (**c**) The distribution of events for each of the two clusters in terms of t_D_ is represented by histograms (bin width 0.02 ms) with peaks at ~70 µs (E1 events) and ~120 µs (E2 events). A gap between the bins for the E2 events has been introduced for better observation of the overlapped distributions. The analyzed events were collected from a single translocation experiment.

The sudden and transient changes in the ionic current elicited by Ang II addition to the *cis* side resemble the electronic signature of peptides interacting with other biological nanopores^11, 14, 16, 43–46^. In-depth analysis of the electronic signature of the transients with the Transalyzer software package^47^ was performed for each individual event in terms of average current change during the transient blockage, <I_B_>, and the dwell time, t_D_. The density plot of the events recorded at −80 mV (Fig. 2a) showed two clusters, a common feature encountered for macromolecules translocated through synthetic and natural nanopores^11, 14, 43, 48^. The presence of two clusters suggests distinct peptide-channel interaction signatures, which may represent opposing orientation of molecules entering the nanopore^7^, folding, oligomerization^11^, binding^46^, or unsuccessful translocation attempts when the molecules only collide with the channel opening^14, 43^. The clusters presented in Fig. 2a are distinct, and hence, were easily separated into two classes of events, named E1 and E2. Further analysis of events belonging to each class showed relatively narrow and symmetric <I_B_> distributions (Fig. 2b), with peaks at ~26 pA (E1 events) and ~10 pA (E2 events).

The <I_B_> values describing either E1 or E2 events represent less than 25% of the absolute value of the open current, which is much smaller than what was measured from experiments investigating peptide or short polynucleotide translocation through other biological nanopores^8, 14, 16, 43, 49^. This discrepancy may be attributed to the structural features of both the channel and peptide. The recently published lysenin structure^35, 36^ indicates a channel length of ~9–11 nm, longer than α-HL and comparable to aerolysin^14^. Lysenin channels have a diameter larger than aerolysin, as indicated by structural data and direct comparison of the open currents through single lysenin channels (as reported in this work) and aerolysin channels in similar conditions^14, 50^. Ang II is a short peptide comprised of only eight amino acids and thus is shorter than many other peptides of known length previously used for translocation through α-HL or aerolysin channels^14^. Therefore, we may reasonably assume that the contour length of the fully stretched peptide is much shorter than the channel’s length and that the volume displaced by each peptide during translocation through the channel is small. Consequently, the peptide interaction with the channels was expected to yield small changes in the ionic open current, and this was experimentally observed. This feature, typical for translocation experiments employing large channels and short peptides^46^, significantly restricted the experimental conditions for investigating Ang II translocation through lysenin nanopores. Traditionally, peptide translocation explorations employ a thorough analysis of the electronic signatures at different voltages and under different ionic conditions^13, 45, 46^. To alleviate problems with recording and analyzing low amplitude transients, short peptide translocation experiments are performed at large holding potentials^13, 46^. Our attempts to reduce the amplitude of the bias voltage to under 40 mV were unsuccessful; the extremely low current blockages, although visible on the traces, made the discrimination between blockages and electrical noise very difficult. A similar situation was encountered when attempting to reduce the electrolyte concentration, which significantly reduced the amplitude of the transient changes of the ionic current. Strong hyperpolarization (potentials under −100 mV) ellicited large atypical fluctuations of the open current, characterized by extended durations. These were presumably attributed to peptide molecules captured by the large fringing electric field being moved towards the nanopore so quickly that they may not properly orient in order to penetrate the pore. Alternatively, they may originate in the gating-like behavior of lysenin channels at large negative voltages, as reported in previous investigations^51^. Therefore we continued our further explorations by using 1 M KCl solutions and holding potentials ranging from −40 mV to −100 mV.

The t_D_ distributions for the two clusters (Fig. 2c) recorded at −80 mV are skewed and non-symmetrical, with peaks at ~70 µs (E1 events) and ~120 µs (E2 events). Unlike the current blockage distributions, that are well separated, we observed major overlapping between the characteristic t_D_s of the two event types. In addition, the E1 events presented a narrow t_D_ distribution while the E2 events spanned a range from 0.02 ms to over 0.4 ms (Fig. 2c). Previous measurements of t_D_s for either peptides or short oligonucleotides translocated through α-HL or aerolysin channels show distributions that follow exponential decays described by characteristic relaxation times as a measure of mean t_D_^11, 13, 14, 16, 43, 46, 49^. Our results may not accommodate such a description since neither of the two event classes can be accurately described as a combination of exponential decays.

**Figure 3:**
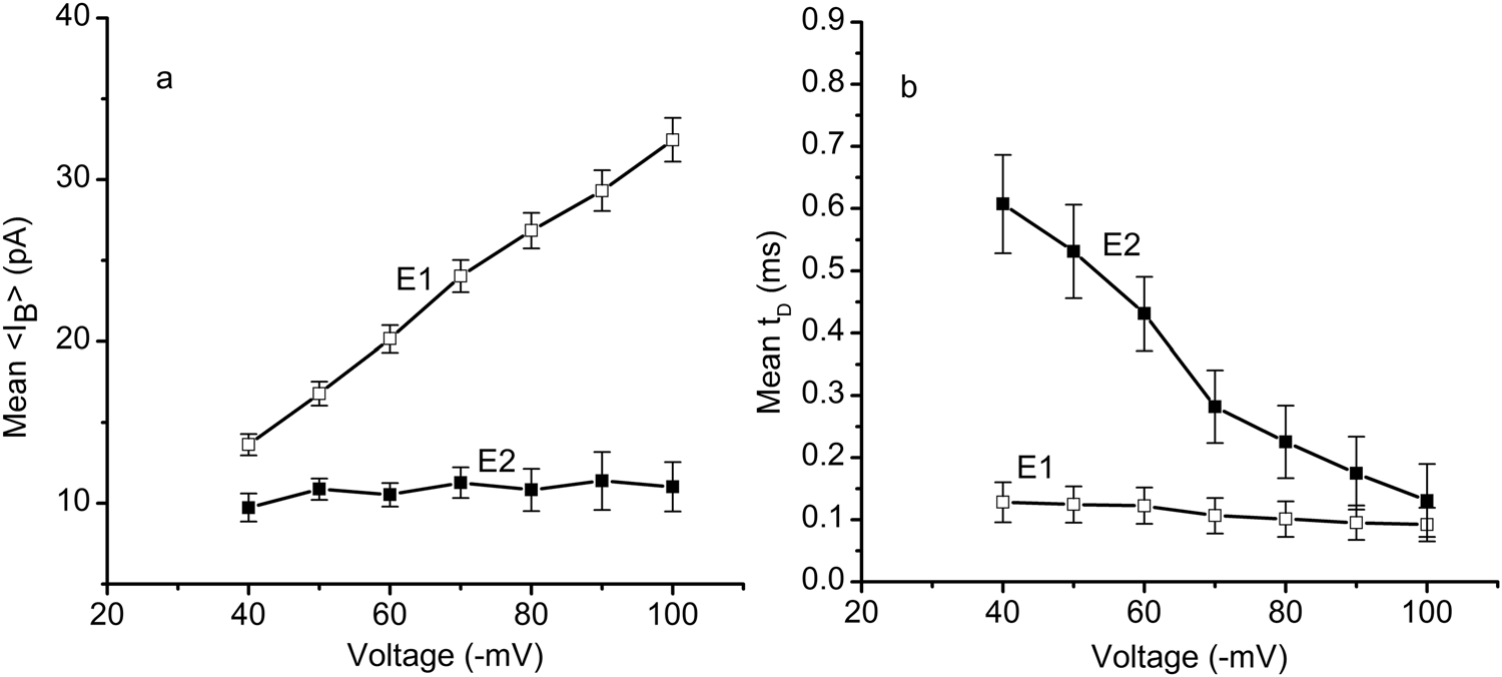
Voltage dependency of the interactions between single lysenin channels and Ang II. (**a**) The mean <I_B_> of events E1 (open squares) followed the applied voltage in a linear manner, as expected for translocations. In contrast, the applied voltage had a much smaller influence on the current blockages characterizing the E2 events (full squares). (**b**) The mean t_D_ of the transient blockages decreased with the applied voltage for both E1 (open squares) and E2 (full squares) events. The data in both panels are represented as mean ± s.d, n = 3; each sample size consisted of at least 2800 events.

For a better interpretation of the experimental results, we performed translocation experiments for multiple transmembrane voltages and analyzed the mean <I_B_ >s and t_D_s for each individual cluster of events recorded at particular voltage conditions. The mean <I_B_ >s measured for E1 events showed a linear change with the applied voltage (Fig. 3a), which is indicative of putative translocation^14^. In contrast, the voltage influence on the mean <I_B_ >s measured for E2 events was minor (Fig. 3a), suggesting failed translocation attempts^14^. Both populations indicated a decrease of the mean t_D_ for increases in the transmembrane voltage (Fig. 3b), but was less pronounced for the E1 events. Since we attributed those events to putative translocations, this seems inconsistent with studies showing that the applied voltage may strongly influence the t_D_^46^. Nonetheless, a recent report on short polynucleotides translocated through a single aerolysin nanopore shows weak voltage dependency for t_D_, which is further diminished at lower pHs^8^. Apparently, short and less charged molecules (like Ang II) present a much smaller voltage influence on the translocation time, suggesting a greater contribution from diffusion on the translocation process. However, at short time scales no transient blockages were observed when the electric field was oriented to drive the cationic peptides away from the channel (Fig. 1c), implying that diffusion alone may not overcome the electrostatic barrier for translocation.

As previously interpreted, we assigned the events characterized by smaller current blockage and longer times to interactions comprising molecules that bump into the pore and diffuse away^14, 43^. In contrast, greater current blockages and shorter times were considered characteristic of putative translocations^14, 43^. Consequently, we concluded that the events E1 are characteristic of translocated molecules and the E2 events represent collisions with the pore. However, we may not completely exclude the possibility of the clustering seen in Fig. 2a as originating from different orientations of the molecules entering the pore^52^, peptide folding, or other complex intermolecular interactions between lysenin channels and peptides^46^. The electronic signature of peptides crossing the nanopores is highly dependent on the nature of both analyte and nanopore, which makes difficult the comparison between translocation experiments for which none of the two is the same.

**Figure 4:**
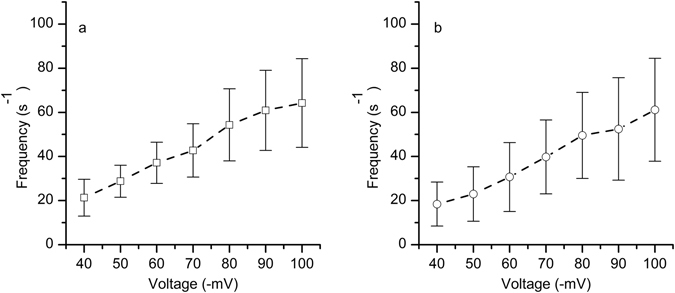
The effect of voltage on event frequency. The frequency of E1 events (**a**) and E2 events (**b**) estimated for a single lysenin channel follows the applied voltage in a quasi-linear manner. The experimental points represents mean ± s.d, n = 3.

Next, we examined the effect of applied voltage on the event frequency for the two distinct populations. As depicted in Fig. 4, the frequency of either E1 or E2 events increased quasi-linearly as the amplitude of the transmembrane voltage increased, which was previously observed for translocation of short nucleotides or peptides^8, 13^. The broad distribution of the experimental frequency values, as inferred from the large error bars, may be explained by a non uniform mixing of the solutions after Ang II addition. We also observed that the event frequency increased with time, which may be explained by an electrically-driven accumulation of peptides in the vicinity of the channel opening. It is worth noticing that the counts were distributed relatively equally between the two populations, irrespective of applied voltages.

### Evidence of translocation: LC-MS analysis

Unlike polynucleotide translocation, which may be simply demonstrated by identification after PCR amplification^29^, proving the translocation of protein molecules across nanopores is challenging to accomplish. Protein and peptide translocation experiments involve sensitive yet sophisticated techniques based on chemiluminescence or fluorescence measurements to provide proof of translocation^11, 49, 53^. Since peptide amplification methodologies are not available, chimeric DNA-peptide molecules have been used to allow identification of translocated products by qRT PCR^42^. Although it is tempting to use such a procedure, the results may suffer from replacing the real analyte with a modified, structurally distinct molecule, possibly becoming the carrier for the analyte itself. Addition of fluorescence tags to the analyte may present similar limitations to correctly interpret the data. Irrefutable evidence of peptide translocation through nanopores requires that there is no modification of the primary molecule; along this line, we chose label-free LC-MS analysis for identification of translocated peptides. Translocation through a single channel may provide numerous translocated molecules, but their precise identification is still beyond what may be achieved by current high sensitivity techniques. To overcome this barrier, one may try to run the translocation experiments over extended time periods to accumulate sufficient molecules for further identification. While this approach may be feasible for synthetic nanopores^53^, it is not necessarily a valid option for biological nanopores inserted into planar lipid membranes, which are fragile, short-lived, and unable to withstand large holding potentials. Alternatively, translocation experiments performed on multiple nanopores at the same time may reduce the time required for the accumulation of detectable amounts of translocated peptide products. To ensure accumulation of sufficient amounts of translocated molecules in this study, we took advantage of the fact that thousands of lysenin channels may be stably inserted into lipid membranes for extended time intervals^54, 55^ and performed translocation experiments employing large populations of channels (~22,700 achieved as described in the methods section).

**Figure 5:**
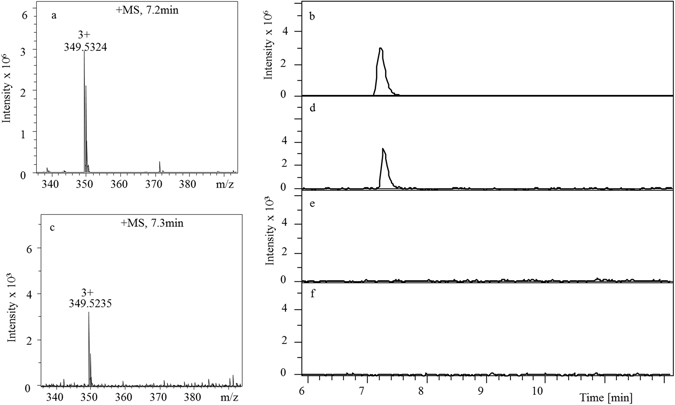
Proof of translocation of Ang II through large populations of lysenin channels inserted into a planar lipid membrane. (**a**) The MS of reference Ang II solution (100 ng) identified the peptide at m/z = 349.5 (z = 3+). (**b**) LC chromatogram of reference Ang II solution. The peptide molecules translocated into the *trans* reservoir at −100 mV for 36 hours were detected and identified by MS (**c**) and LC (**d**). Application of a +100 mV voltage (**e**) or channel blockage by chitosan (**f**) did not allow LC detection of Ang II into the *trans* reservoir.

After Ang II was added to the *cis* reservoir (10 µg mL^−1^ final concentration) at −100 mV for ~36 hours, solution from the *trans* reservoir was sampled for further analysis. LC-MS showed the presence of Ang II in a standard sample and in the solution sampled from the *trans* reservoir (Fig. 5a–d), suggesting the passage of Ang II through the membrane containing a large population of lysenin channels. The amount of translocated Ang II (~ 0.8 ng) estimated from a MS-LC calibration curve of standard amounts has been used to compute a translocation frequency of ~1,100 events/s. This is larger than the average value estimated from single channel experiments at the same voltage (see Fig. 4). However, taking into account the large variability of the frequency data, we may consider the two measurements in satisfactory agreement. It is interesting that a better frequency match will be obtained by considering all events as representing translocations but this raises serious questions about the fundamentally different effects that voltage may have on the two event types. Either way, more assurance of peptide translocation through the channels was needed since it has been reported that Ang II could interact with artificial lipid membranes^56, 57^. Earlier investigations showed an increase of the lipid bilayer conductance upon peptide addition at a high concentration, which was assumed to originate in pore formation^56^, while later reports point out a sustained adsorption at the membrane surface^57^. Although none of those studies showed that Ang II crosses the lipid membrane spontaneously, such a potential issue may compromise our interpretation with regards to putative translocation through lysenin channels. To examine if the translocation of Ang II was facilitated by other peptide-membrane interactions, we implemented multiple control experiments designed to eliminate concerns with regard to potential leakage of membranes supporting a large population of channels for extended time. After achieving a similarly large population of inserted lysenin channels, Ang II was added to the *cis* side while applying a positive transmembrane voltage of 100 mV. At this voltage some of the channels may undergo voltage-induced gating, but this feature is seriously diminished at high salt concentrations and in congested conditions^54, 55^. Thus, many channels were assumed to remain open. LC analysis showed no detectable peptide in the sample collected from the *trans* reservoir (Fig. 5e), which indicates the necessity of proper orientation of the electric field for Ang II to cross the membrane.

One may argue that in the above experimental conditions the opposite electric field may also prevent translocation through a leaky membrane or that lysenin channels may present voltage gating by adopting sub-conducting states^41^ characterized by a reduced diameter of the conducting pathway, which is incompatible with translocation of large molecules. Therefore, we performed a similar experiment in which the membrane was biased by −100 mV (to promote translocation) but the lysenin channels were exposed to chitosan, an irreversible blocker of lysenin channel’s conductance^58^. Again, the LC analysis of samples extracted from the *trans* reservoir did not show the presence of Ang II (Fig. 5f). Although it is possible that spontaneous translocation of Ang II occurred at levels below the LC-MS detection limit, it is evident that open lysenin channels inserted into the membrane were responsible for mediating the electrophoretically-driven Ang II translocation observed in this study. Together with the electronic signature recorded on single lysenin channels in the presence of peptide, our work demonstrates that lysenin channels allow the peptides to cross the membrane through the nanopore’s conducting pathway.

## Conclusions

We have successfully demonstrated that lysenin channels inserted into lipid membranes facilitate the translocation of peptide molecules electrophoretically driven by electric fields. This study adds lysenin to the short list of promising pore-forming proteins suitable for developing nature-inspired single molecule sensing and characterization devices. The channel’s large and uniform diameter may accommodate molecules that are too large to be translocated by other biological nanopores, therefore extending the sensing capabilities at nanoscale for other biomolecular analytes. Further channel engineering by chemical modifications and site-directed mutagenesis for implementation of improved or even novel sensing capabilities is now possible owing to recent structural data of the assembled pore^35, 36^. In addition, lysenin channels present unusual regulatory mechanisms by physical and chemical stimuli such as voltage or ligands^41, 54^. The external modulation of the conducting state may be further used for controlled transport of bioactive molecules through natural and artificial lipid membranes, temporary cell permeabilization, drug delivery systems, intelligent switches, and bioelectronics.

## Methods

### Bilayer Lipid Membrane preparation and channel insertion

**Figure 6:**
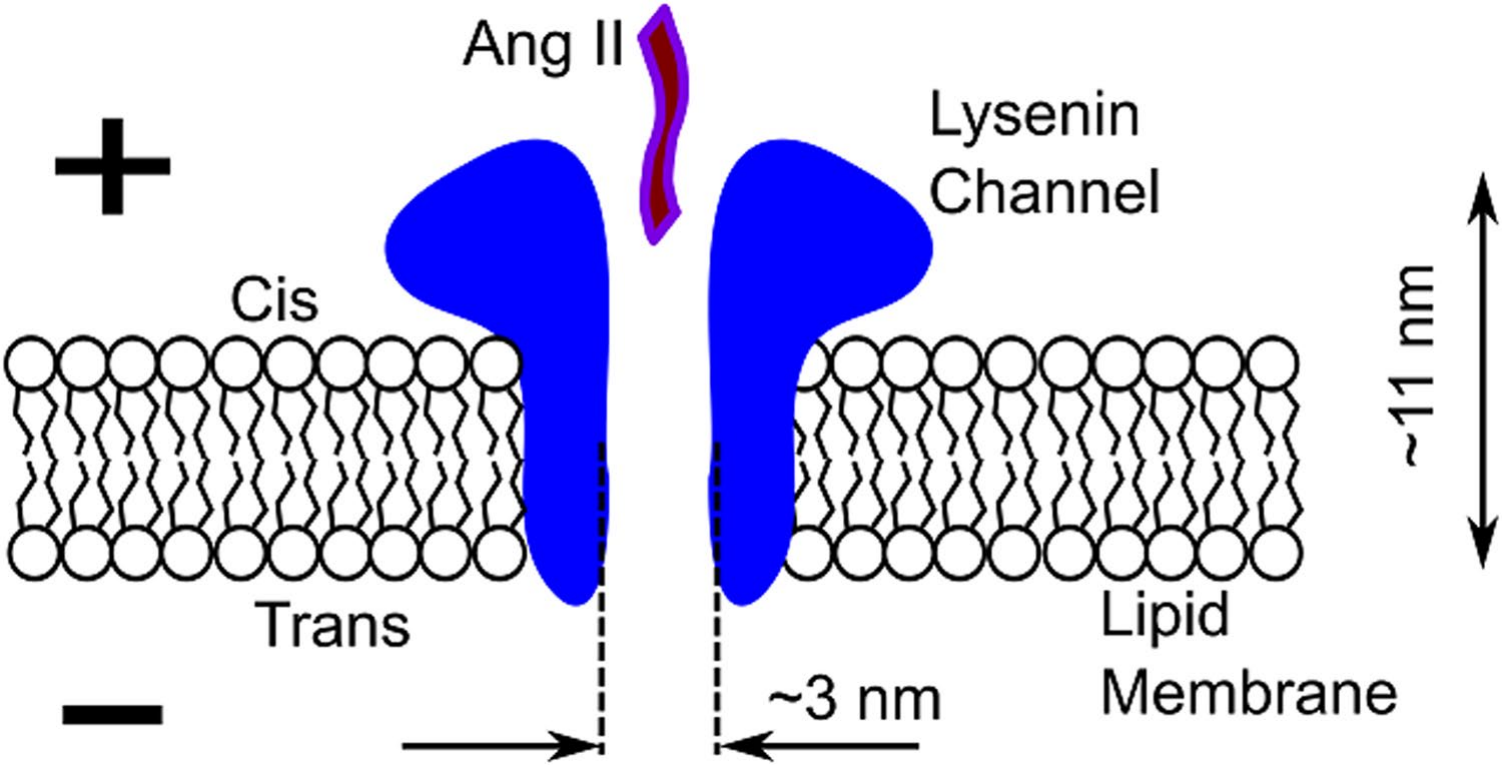
Simplified sketch of the experimental setup for Ang II translocation. Single lysenin channels inserted into planar bilayer lipid membranes biased by transmembrane voltages may facilitate the electrophoretically driven translocation of macromolecules through the large conducting pathway. The channel’s dimensions are from ref. 35.

The membrane was formed in a classic bilayer setup^40, 55, 59^ consisting of two electrolyte-filled reservoirs made of polytetrafluoroethylene (PTFE) and separated by a thin PTFE film (120 μm thickness) in which a small hole (~60 µm diameter) was created by using an electric spark. Each reservoir was filled with up to 1 mL of 1 M KCl solution buffered with 10 mM Tris and 1 mM EDTA at pH 6.9. The stock lipid mixture contained 4 mg diphytanoyl phosphatidylcholine (Avanti Polar Lipids), 2 mg sphingomyelin (Avanti Polar Lipids), and 2 mg cholesterol (Sigma-Aldrich) dissolved in 200 µL n-decane. The electrical connections were established with two Ag/AgCl electrodes immersed in the electrolyte solutions on both sides of the membrane and connected to an Axopatch 200B amplifier feeding a Digidata 1440 A Digitizer (both from Molecular Devices). The digitized signal was recorded with the Clampex 10.5.2.6 software package (Molecular Devices) for further analysis, as required by each experiment. After bilayer formation, 1 μl of 100 nM lysenin stock solution was added to the *cis* (ground) reservoir under continuous stirring with a Bilayer Magnetic Stirrer (Warner Instruments). A −60 mV bias potential was applied and channel insertion recorded. After channel insertion, the reservoir was flushed with 30 mL lysenin-free buffered electrolyte. A simplified diagram of the experimental setup is presented in Fig. 6.

### Peptide translocation through single lysenin channels

Ang II was added to the *cis* reservoir at a final concentration of 1 μg mL^−1^, followed by stirring for ~10 seconds. The transient events were recorded with the variable-length protocol at a 4 µs sampling time, 1 ms pre- and post-trigger length, 10 kHz hardware filter, and were saved for analysis with Clampfit 10.2 (Molecular Devices), Transalyzer^47^, Matlab (Mathworks), and Origin 8.5 (OriginLab Corporation) software packages. The dwell time was calculated as the Full Width Half Maximum for each individual event selected for analysis, and the current amplitudes were calculated as the average level between the first and the last local minima of each event^47^. This procedure provided the best separation between the E1 and E2 events.

### Insertion of large populations of lysenin channels

**Figure 7:**
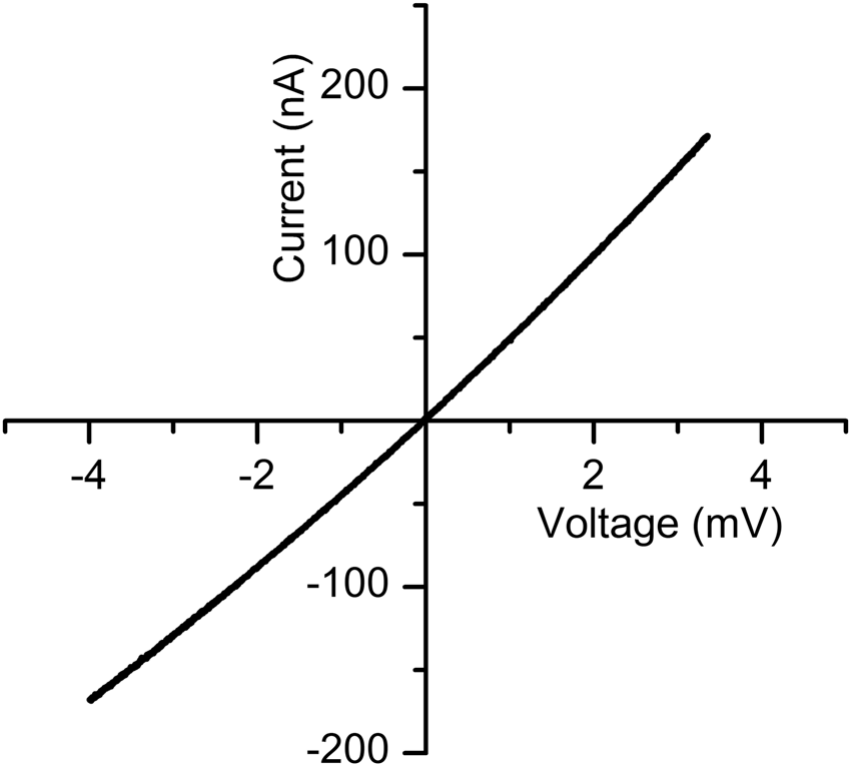
The I-V plot for large populations of lysenin channels inserted into the bilayer lipid membrane. The conductance calculated from the slope of the curve was used to estimate the number of inserted channels.

**Figure 8:**
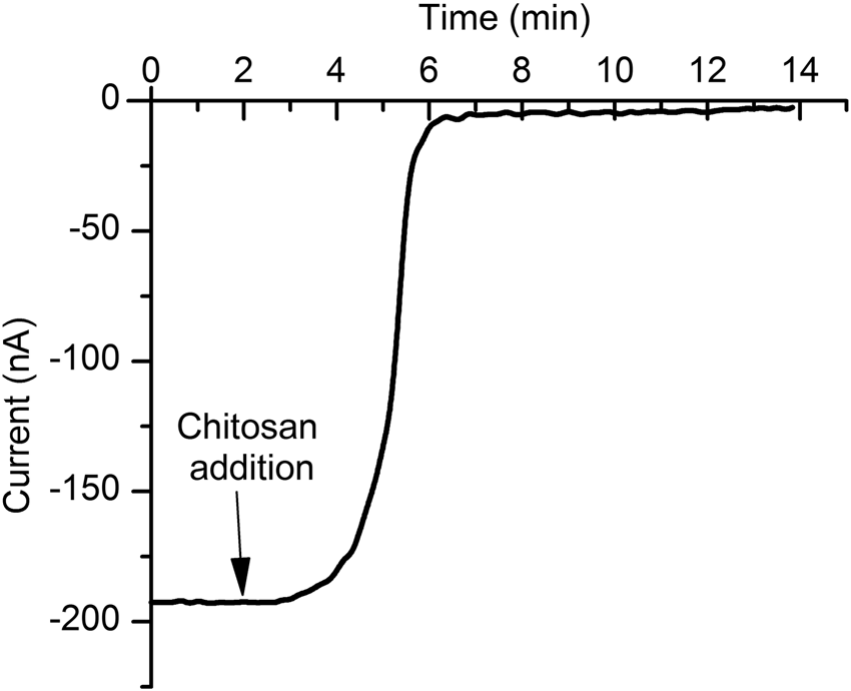
Blockage of lysenin channels by chitosan. Chitosan addition induced a sustained and irreversible decrease of the macroscopic conductance at −4 mV transmembrane potential, indicative of channel blockage.

The experimental protocol for insertion of multiple channels into the bilayer was similar to that for single channels but involved a larger diameter for the hole in the PTFE film (~180 µm in diameter, to accomodate larger populations of channels). A higher amount of lysenin was added to the *cis* reservoir (up to 20 µL of 1 µM), and a lower electrolyte volume in the *trans* reservoir (100 µL) was used. The total number of inserted channels was sequentially increased by successive additions of lysenin until a steady ionic current of ~ −180 nA at −4 mV transmembrane voltage was achieved, and then the buffered electrolyte was exchanged to remove lysenin from the bulk solution. The number of channels residing in the membrane (~22,700) was estimated from the ratio between the total membrane conductance determined from the I-V curve recorded for a narrow voltage range (Fig. 7) and the individual channel conductance (~2.03 nS) determined from single channel insertion experiments as depicted in Fig. 1. Multichannel translocation and control experiments comprised addition of 10 µg of Ang II to the *cis* reservoir (containing 1 mL ionic solution) and application of specified voltages which were provided by a custom made voltage source (to avoid long term overloading of the electrophysiology amplifier) for extended time (36 hours). After all components were added to the solutions, the reservoirs were covered with thin silicone films to avoid evaporation. With all of the precautions, less than 10% of the membranes survived for the long duration required by experiments (most ruptured, but some also re-assembled as multilayers). The integrity of each bilayer was checked at the end of each experiment by estimating their conductance from I-V plots, and those presenting large deviations from the initial values have not been considered for further analysis. Our first control comprised application of a positive transmembrane potential (+100 mV) that produced an electrophoretic force which opposed translocation. For the second control, we irreversibly blocked the lysenin channels before Ang II addition by adding to the *cis* reservoir 5 µL of 0.1% (m/v) chitosan solubilized in 0.1 M acetic acid. Chitosan addition ellicited a fast decrease of the ionic current (Fig. 8) at −4 mV transmembrane voltage, indicative of channel blockage. The small, residual current recorded after the chitosan-induced blockage was negligible in comparison to the initial open current. Its source is unknown, but may originate from channels remaining in the open state, leakage through partially blocked channels, or leaks through the membrane. Nonetheless, this leakage did not promote translocation of detectable amounts of Ang II, as shown by results of the LC experiments. After channel blockage and Ang II addition, the reservoirs were covered and the membrane biased by −100 mV for 36 hours.

### LC-MS analysis

Samples from the *trans* reservoir (where the peptide was translocated to) were carefully extracted without breaking the bilayer membrane. Standard samples containing between 0 ng and 100 ng of Ang II were prepared for the standard curve, which was constructed by integrating the areas under the LC peaks. The translocation of Ang II through lysenin channels was confirmed using a Bruker maXis Quadrupole-Time-of-Flight (Q-TOF) mass spectrometer equipped with an Electrospray Ionization (ESI) source (Bruker Daltonics). ESI-Q-TOF was coupled with a Dionex Ultimate 3000 LC system (Thermo Scientific), and chromatographic separation was performed on a Phenomenex C18 column (150 × 2.1 mm, 4 µm, Phenomenex). The samples were placed in an autosampler at 4 °C and each sample was injected onto the column. The LC elution mobile phases consisted of solvent A (5% acetonitrile and 0.1% formic acid in water) and solvent B (0.1% formic acid in acetonitrile). The elution started at 0% B, held at this percentage for 9 minutes, increased to 25% B over 10 minutes, further increased to 60% B over an additional 11 minutes, and then was kept at this percentage for 21 minutes. The LC flow rate was maintained at 200 µL min^−1^ and the temperature of the column was maintained at 40 °C during the analysis. Mass spectrometry analysis was performed in positive ion mode with a spray voltage of 3000 V, an endplate offset of −500 V, a nebulizer gas pressure of 1.5 bar, a dry gas flow rate of 8.0 l min^−1^, and a dry gas temperature of 200 °C.
